# Anterolateral Radical Debridement and Interbody Bone Grafting Combined With Transpedicle Fixation in the Treatment of Thoracolumbar Spinal Tuberculosis

**DOI:** 10.1097/MD.0000000000000721

**Published:** 2015-04-10

**Authors:** Zhaohui Cheng, Jian Wang, Qixin Zheng, Yongchao Wu, Xiaodong Guo

**Affiliations:** From the Department of Orthopaedics, Union Hospital, Tongji Medical College, Hua-zhong University of Science and Technology, People's Republic of China (ZC, JW, QZ, YW, XG); and Department of Orthopedic Surgery, The Second Affiliated Hospital, School of Medicine, Zhejiang University, People's Republic of China (ZC).

## Abstract

This retrospective cohort study was conducted to evaluate the clinical outcomes of radical anterolateral debridement and autogenous ilium with rib or titanium cage interbody autografting with transpedicle fixation for the treatment of thoracolumbar tuberculosis.

Spinal tuberculosis operation aims to remove the lesions and necrotic tissues, remove spinal cord compression, and reconstruct spinal stability. However, traditional operation methods cannot effectively correct cyrtosis or stabilize the spine. In addition, the patient needs to stay in bed for a long time and may have many complications. So far, the best surgical method and fixation method for spinal tuberculosis remain controversial.

There were a total of 43 patients, 16 involving spinal cord injury, from January 2004 to January 2011. The patients were surgically treated for radical anterolateral debridement via posterolateral incision and autogenous ilium with rib or titanium cage interbody autografting and single-stage transpedicle fixation. All the patients were followed up to determine the stages of intervertebral bone fusion and the corrections of spinal kyphosis with the restoration of neurological deficit.

The erythrocyte sedimentation rate (ESR) of these patients decreased to normal levels for a mean of 2.8 months. The function of feeling, motion, and sphincter in 16 paraplegia cases gradually recovered after 1 week to 3 months postoperatively, and the American Spinal Injury Association scores significantly increased at the final follow-up. Intervertebral bone fusions were all achieved postoperatively. No internal fixation devices were loose, extracted, or broken. There was no correction degree loss during the follow-up.

The method of radical anterolateral debridement and autogenous ilium with rib or titanium cage interbody autografting and single-stage transpedicle fixation was effective for the treatment of thoracolumbar tuberculosis, correcting kyphotic deformity, and reconstructing spinal stability, obtaining successful intervertebral bony fusion and promoting the recovery of paraplegia. These results showed satisfactory clinical outcomes.

## INTRODUCTION

Spinal tuberculosis is one of the most common extrapulmonary tuberculosis,^1^ accounting for the top tuberculosis of total bones and joints. The morbidity of this tuberculosis is high, often leading to collapse, wedging, and cyrtosis in the vertebral body of the invaded segment. In addition, spinal tuberculosis forms abscesses, sequestrum, and tuberculous granulation tissue, which enter the spinal canal to compress the spinal cord, causing nerve damage or even paraplegia. Spinal tuberculosis is a heavy burden on the patients and their families, and it is a big problem of the society. Based on combination chemotherapy, active surgical treatment has been accepted and can effectively shorten the treatment cycle, promote tuberculosis cure, reduce morbidity, and improve the quality of life. In this paper, combining with transpedicle fixation, anterolateral radical debridement and grafting of interbody bones were employed in 43 patients’ treatment with thoracolumbar tuberculosis from January 2004 to July 2011. The cases were followed up for 1.0 to 3.5 years. This method achieved good results, and the report is as follows.

## OBJECTIVES AND METHODS

### General Information

A total of 43 patients (males: 26; females: 17) underwent surgery for thoracolumbar tuberculosis from January 2004 to July 2011. The patients’ age varied from 21 to 53 years, with an average age of 32.3 years. Written informed consent was obtained from all patients and the study protocol was approved by the Ethics Committee of the Union Hospital, Tongji Medical College, Huazhong University of Science and Technology. Of these cases, 21 were thoracic spine and 22 were lumbar spine, and the involved segments were from thoracic 3 to lumbar 5. The clinical manifestations included tuberculosis poisoning symptoms, such as lower back pain, restricted movement, low fever, loss of appetite, emaciation, and different degrees of cyrtosis of thoracic and lumbar spine.

A total of 16 patients underwent spinal cord compression: according to the American Spinal Injury Association (ASIA) classification, 11 cases were at grade C, and 5 cases were at grade D. Before surgery, all the patients underwent X-ray, computed tomography and magnetic resonance imaging (MRI) examination. The results showed affected vertebral destruction and collapse, and narrowed or disappeared intervertebral space. A total of 36 cases involved single segments, while 7 cases involved multiple segments. Among these cases, 16 with incomplete paraplegia showed different degrees of space occupying the compressed spinal cord, including caseous material, inflammatory granulation tissue, and sequestrum. The preoperative Cobb angle of cyrtosis was 9° to 54°, with an average of 39.2°. All the patients underwent 1-stage lateral anterior debridement with autografting, rib or titanium mesh implantation in the intervertebral defect area combined with posterior internal fixation with a pedicle screw system. After the operation, the patients underwent anti-tuberculosis treatment for >1 year. The spinal tuberculosiss in this group were all primary, and the lesion segment did not have any history of surgery. After examination, active pulmonary tuberculosis was excluded. Preoperative anti-tuberculosis drug had an obvious curative effect.

### Preoperative Preparation

The patients underwent conventional chest radiograph and related biochemical examination. Pulmonary tuberculosis was excluded. Triple therapy with isoniazid, rifampin, and ethambutol or quadruple therapy with streptomycin in addition to the former 3 drugs was used for 2 to4 weeks. All tuberculosis poisoning symptoms were under control, and the blood pressure and blood sugar levels were within the scope of operation. With strengthened nutrition supporting therapy, hypoalbuminemia and anemia were corrected. When there was improvement in anemia and the liver and kidney function were normal, the operation was performed. In general, preoperative blood sedimentation should not be >40 mm/h, while hemoglobin (Hb) should not be <100 g/L.

### Surgical Methods

Anesthesia was administered via tracheal intubation. An incision was made at the posterior midline. A pedicle screw was fixed in the external vertebral bodies with the normal intervertebral spaces at the 2 ends of the lesions, with 1 to 2 pairs, respectively, at the top and the bottom. A Luque rod was used to correct cyrtosis as far as possible to restore the original spine radian before closing the incision. On the side with more serious bone destruction and more abscesses, another incision was made for debridement, namely through the paraspinous longitudinal incision, and the rib-transverse process was resected to expose thoracic lesions front the lateral anterior. Through the kidney incision or V-shape incision, the thoracic lumbar segments or lumbar spinal lesions were exposed. Pus was completely cleaned, and the tuberculosis materials, such as the sequestrum and necrosis of intervertebral disc, were removed. The contralateral vomica was stretched through the intervertebral disc and destructed vertebral bodies as far as possible to move out the contralateral pus. The rib cut, autogenous iliac or titanium mesh were implanted into the vertebral defect to stabilize and repair the spine.

### Postoperative Treatment

The patients were administered conventional antibiotics for 5 to 7 days until the hemogram was normal. Then, 72 h after surgery, according to the condition of drainage, the drainage tube was removed; the patients were directed to stay in bed for 3 weeks postoperatively and then wear the thoracolumbosacral orthosis for 4 to 6 months. From the first day after operation, anti-tuberculosis drugs, such as isoniazid, rifampin, and ethambutol, were administered and were continued to be used for more than 1 year. X-ray was reviewed once a month until complete bone graft fusion, after which radiography was conducted every 3 months or half year. Secretion and antrum at the incision, looseness and fracture of the internal fixation, and loss of deformity correction were observed. The recovery status of neural function in patients with nerve damage recovery was also determined. The liver and kidney function as well as blood sedimentation were reviewed periodically, and the side effects of anti-tuberculosis drugs were closely observed. Treatment was adjusted when necessary.

## RESULTS

Intraoperative complications, such as spinal cord, and nerve and lung damage, were not observed. According to the pathology results, specimens from postoperative debridement were confirmed as tuberculosis. Approximately 1 to 3 weeks after the operation, the symptoms significantly reduced, and the entire condition of the body was better. A total of 43 cases in this group were followed up for 1.0 to 3.5 years, with an average of 21 months. Local recurrence of spinal tuberculosis and fistula formation was found in 3 cases. The wound was healed through fistula curettage and dressing change. The remaining patients had both incisions healed without skin flap necrosis, and the lower back pain disappeared.

**FIGURE 1:**
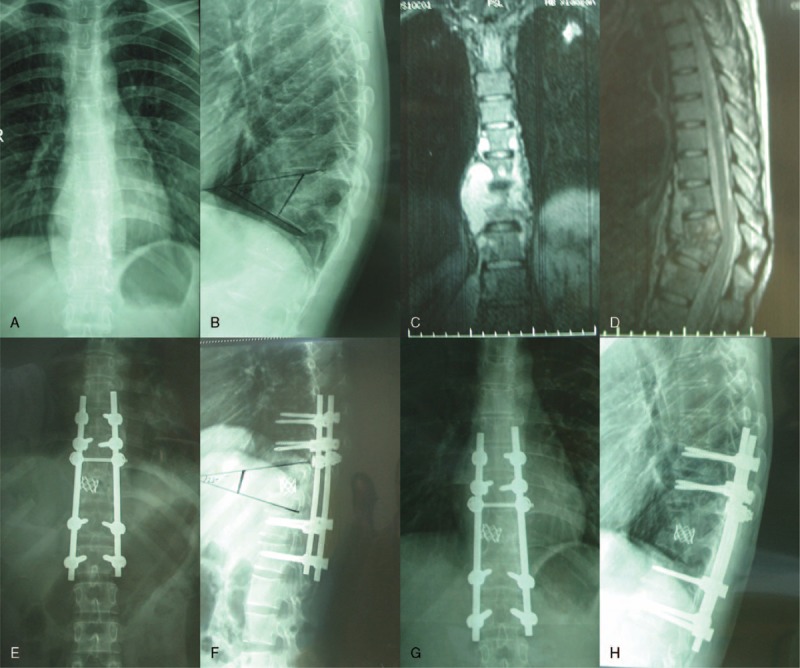
A 25-year-old female patient (a–h), preoperative X-ray (a, b) and MRI (c, d) shows T8, T9 vertebral body bone destruction with cyrtosis, compressed spinal dura mater, and paravertebral abscess. Postoperative 1 week, X-ray (e, f) show vertebral body height corrected with Cobb angle. Postoperative 1 year, X-ray (g, h) showed a good fixed position. MRI = magnetic resonance imaging

**FIGURE 2:**
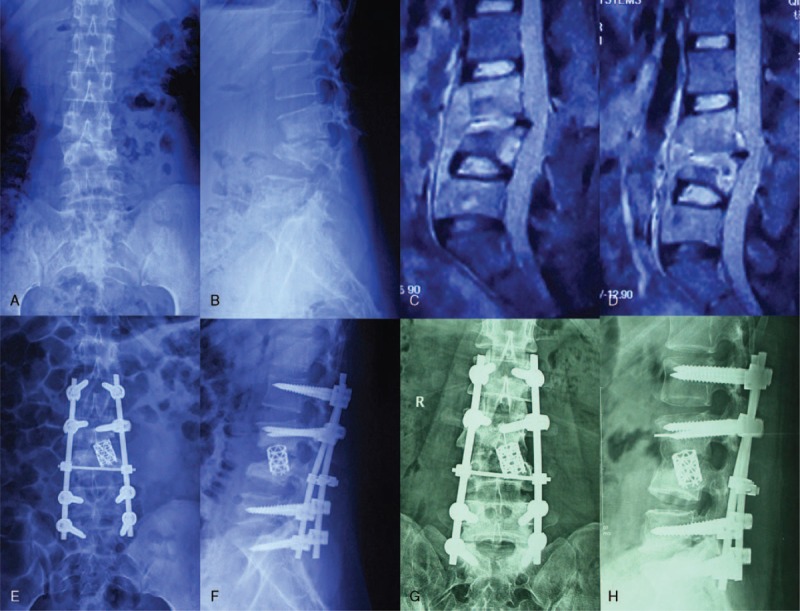
A 35-year-old female patient (a–h), preoperative X-ray (a, b) and MRI (c, d) shows L3, L4 vertebral body bone destruction with cyrtosis, compressed spinal dura mater, and paravertebral abscess. Postoperative 1 week, X-ray (e, f) show vertebral body height corrected with Cobb angle. Postoperative 1 year, X-ray (g, h) showed a good fixed position. MRI = magnetic resonance imaging

Erythrocyte sedimentation rate (ESR) gradually declined within 3 to 6 weeks postoperation. The ESR decreased to a normal level after an average of 2.8 months. Within the first week postoperatively, X-ray was used to review. The positions of bone graft or titanium mesh and their internal fixation were good in all the patients. During the follow-up, all the lesions were well repaired, and the bone graft or titanium mesh had a good bone fusion with the surrounding tissue, with a fusion rate of 100%. The fusion time for the single segment was 4 to 6 months, while that for multiple segments was 6 to 12 months. There was no obvious loosening or prolapsed internal fixation, nor were there broken or loose screws. The Cobb angle of cyrtosis at postoperative 1 week was 8.6° (5–13.8°). The average correct angle was 30.6°, and the correct rate was 78%. No obvious correction degree loss was detected during the follow-up period. A total of 16 patients with incomplete paraplegia had gradually recovered their sense, myodynamia and sphincter function in postoperative 1 week to 3 months by the last follow-up, and except for 1 patient whose ASIA score was at D level; nerve functions recovered in all the other patients (Table 1). Typical cases are shown in Figures 1 and 2.

**TABLE 1:**
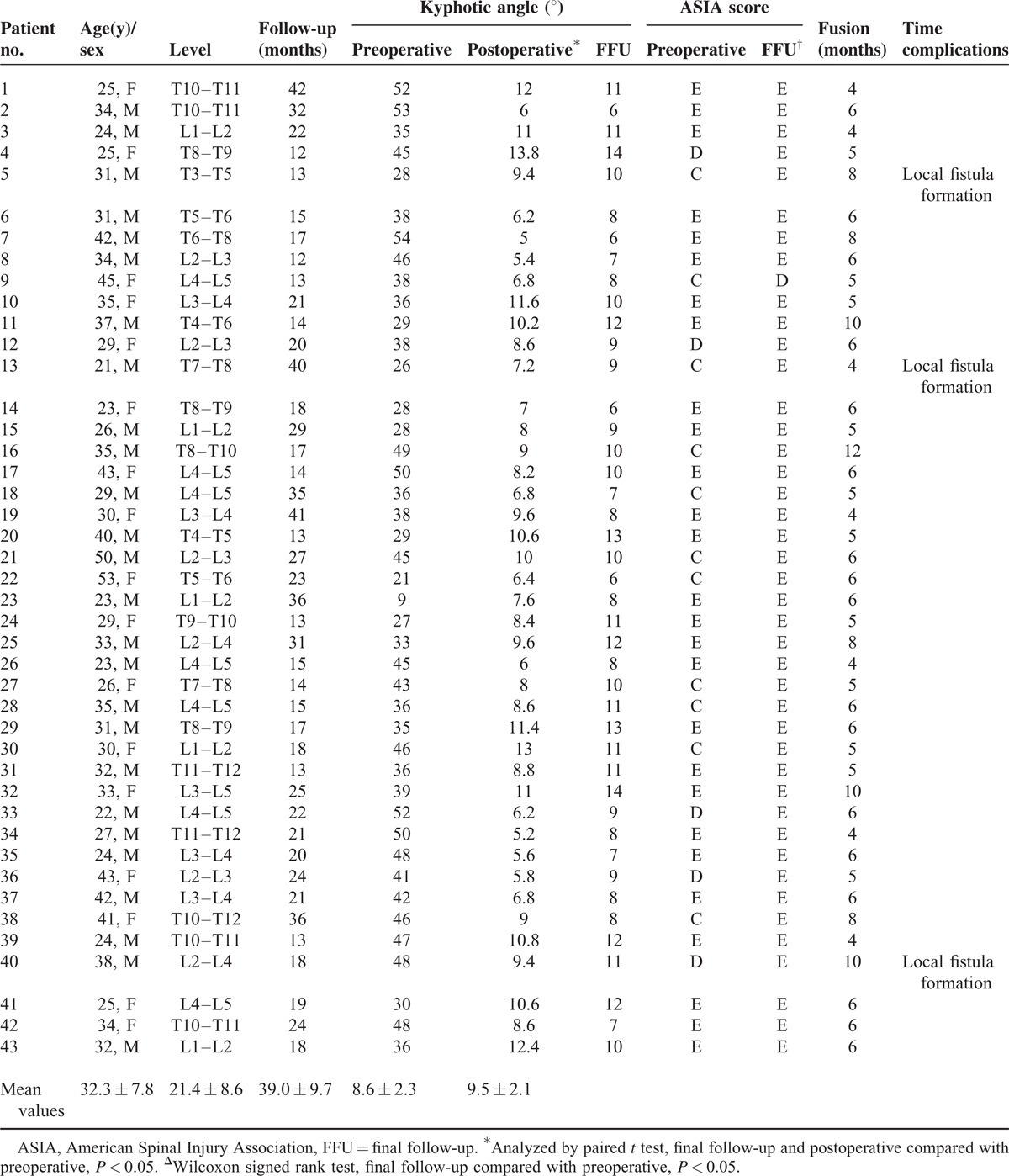
Summary of Clinical Data on All Patients

## DISCUSSION

### Surgery for Spinal Tuberculosis

Spinal tuberculosis operation aims to remove the lesions and necrotic tissues, remove spinal cord compression, and reconstruct spinal stability. However, traditional operation methods cannot effectively correct cyrtosis or stabilize the spine. In addition, the patient needs to stay in bed for a long time and may have many complications. This method even leads to the movement and absorption of the graft, thus causing the progression of the cyrtosis, even leading to late-onset paralysis. Especially when the graft is across 2 intervertebral discs, most patients require reoperation treatment supplemented by internal fixation.^2^ In recent years, an internal fixation technique has been increasingly used in spinal tuberculosis operations. The characteristics of internal fixation are as follows:

1. It can shorten the postoperative bed time and is easy for nursing care and rehabilitation.
2. It increases the rate of bone graft fusion.
3. It is stable at lesion site, facilitating tuberculosis control; the postoperative recurrence rate is decreased; and it can correct spinal deformity.

The best surgical method and fixation method of spinal tuberculosis remain controversial, however.^3,4^ tuberculosis lesions frequently involve the anterior and middle column of the spinal column, and anterior or anterolateral radical debridement is intuitive and thorough. Especially with nerve damage, multiple segmental involvement, and serious abscess formation, if the interbody bone grafting is combined with posterior transpedicle fixation, an adequate supporting function can be provided for the anterior column, thereby preventing implant-related complications and effectively correcting cyrtosis or preventing its development,^5,6^ which is advantageous to the bone graft fusion. Therefore, for spinal tuberculosis, anterolateral radical debridement and interbody bone grafting combined with posterior transpedicle fixation are favored by the scholars.^7–13^ In this study, with double incisions and double approaches, the patients were given anterolateral radical debridement and interbody grafting of autologous bone or titanium mesh combined with posterior transpedicle fixation. The patients were followed up and reported satisfactory clinical effects.

### Advantages and Disadvantages of Radical Anterolateral Debridement and Interbody Bone Grafting With Posterior Transpedicle Fixation

The double incisions and double approaches have obvious advantages, which are as follows:

1. Exposing the incision of anterolateral vertebral body can avoid opening the chest and abdominal cavity, can better reveal the thoracic and lumbar tuberculosis lesions for debridement and anterolateral decompression, and intervertebral bone graft surgery. Compared with the approach via thoracic cavity and other approaches, the probability of surgical trauma and postoperative complication is small.
2. Thoracic and lumbar transpedicle grafting is mature, and mechanical stability is good. Debridement and interbody bone grafting with transpedicle fixation can be completed in 1 stage. Because the internal fixation is located at the rear, debridement can be more thorough because it does not need to consider the location of internal fixation.
3. The metal implant does not directly contact tuberculosis lesion by using internal fixation outside the lesions, which can reduce the risk of lesion movement, can reduce the delay in recovery due to the contact, and can prevent the spread of infection; Also internal fixation outside the lesion is not affected by tuberculosis recurrence for it can be taken out in the stage II operation. In addition, compared with the internal fixation on the vertebral side, this method does not need to expose the normal vertebral body at both ends of the lesions and does not expose the normal tissues surrounding the lesions. Fewer ribs need to be removed, and fewer vascular segments between ribs need ligation. The removal of the normal intervertebral disc on both ends of the lesions can be avoided. The implant bone segment is short, and the segment that needs fusion is also reduced.
4. Three-column transpedicle fixation provides more strong and reliable fixation for the fusion segment, which can effectively restore the normal physiological curvature of the spine, correct cyrtosis, and avoid the late-onset spinal cord damage that is caused by angle loss and aggression of the cyrtosis.^14^ The stability of the spinal sagittal plane is reconstructed at the same time, providing a good mechanical environment for the standstill of tuberculosis lesions and spinal fusion, thereby reducing the recurrence of spinal tuberculosis.
5. For multiple segmental lesions, 2 or more vertebral bodies are required to be fixed at sites above and below the lesions to fully recover the mechanical properties of the spine. In contrast, posterior fixation is much easier, and the postoperative load is dispersed to several pedicle screws, which can effectively avoid complications, such as loosening or prolapsed internal fixation, especially for elderly patients with severe osteoporosis.

However, radical anterolateral debridement and interbody bone grafting with posterior transpedicle fixation also have shortcomings, which are as follows:

1. Due to 2 incisions, the skin flap blood supply between the incisions should be monitored.
2. The contralateral lesion cannot be exposed in the straight view, making the complete removal difficult.
3. Some of the posterior ribs need to be cut for the entire approach, and blood vessels that supply the spinal cord need ligation. One-side vascular ligation on 2 pairs of blood vessels cannot affect the spinal cord blood flow, but that on 3 pairs or more will affect the spinal cord blood flow. Therefore, we should be careful when multiple segmental vertebral bodies are involved in thoracic spinal tuberculosis.

### Surgical Indications and Curative Effect

An analysis of the results of this study indicates the following:

1. The lesions should be located in the thoracic and lumbar spine, and the affected segments should be no more than 3.
2. The lesions should be concentrated on one side.
3. Patients should be without active tuberculosis, and the body should be in good condition. Surgical timing is very important. Patients should receive at least 2 to 4 weeks of effective anti-tuberculosis drugs therapy preoperatively. Only when the blood sedimentation is markedly reduced (usually <40 mm/h), tuberculosis poisoning symptoms are reduced, basic normal liver and kidney function is normal and anemia is improved (Hb > 100 g/L) can the surgery be performed.

The cases in this study were given anterolateral radical debridement and interbody bone grafting with posterior transpedicle fixation, underwent spinal cord compression removal, and corrected cyrtosis with a correction rate of 78%. Spinal stability is effectively reconstructed. With anti-tuberculosis drugs, the symptoms, and general condition of the patients improved gradually as observed in the follow-up. The ESR gradually decreased and returned to normalcy. At the FFP, all the bone grafts achieved bony fusion, and no obvious correction loss and no graft or internal fixation-related complications occurred. Therefore, we believe that thoracolumbar tuberculosis treated with anterolateral radical debridement and interbody bone grafting with posterior transpedicle fixation can obtain satisfactory clinical effects. Spinal anterior or posterior fixation can be used in the treatment of thoracolumbar tuberculosis^15–18^; however, anterior fixation has limited function for cyrtosis correction and is prone to develop postoperative correction loss. Recently, Wang et al^19^ reported cases undergoing anterior debridement and internal fixation with titanium mesh to treat thoracic and lumbar spinal tuberculosis; all the patients underwent titanium mesh sink and secondary correction loss in the long-term follow-up. Ma et al^14^ reported 157 cases of spinal tuberculosis patients with surgical treatment. Compared with the curative effects of anterior fixation (74 cases) with posterior fixation (83 cases), anterior and posterior fixation are both believed to achieve good clinical effects, but posterior fixation has more advantages in cyrtosis correction and stability maintenance. Combined with the cases in this study, it is believed that based on the strictly mastered indications and better operation time, anterolateral radical debridement and interbody bone grafting with posterior transpedicle fixation for treating thoracolumbar tuberculosis can obtain satisfactory clinical effects. It needs to be emphasized that the basis of spinal tuberculosis treatment is still the preoperative and postoperative standardized anti-tuberculosis treatment.
